# Correlation Between the Thickness of Anterolateral Ligament and Lateral Collateral Ligament of the Knee

**DOI:** 10.7759/cureus.12668

**Published:** 2021-01-12

**Authors:** Prashant Chaware, John A Santoshi, Aditi Chaurasia, Madhuri Parija, Urvashi Singh, Bertha A Rathinam

**Affiliations:** 1 Anatomy, All India Institute of Medical Sciences, Bhopal, IND; 2 Orthopaedics, All India Institute of Medical Sciences, Bhopal, IND; 3 Radiology, All India Institute of Medical Sciences, Bhopal, IND; 4 Orthopaedics, Kasturba Hospital Bhel, Bhopal, IND

**Keywords:** anterolateral ligament, acl, anterolateral complex, lateral collateral ligament, anterolateral rotatory instability

## Abstract

Background

Persistent anterolateral rotatory instability (ALRI) following the anterior cruciate ligament (ACL) reconstruction has led to a renewed interest in defining the role of anterolateral complex (ALC) of the knee.

Methods

We explored the anterolateral corner of 34 cadaveric knees to define the anterolateral ligament (ALL) in all its dimensions and measured the thickness of lateral collateral ligament (LCL) at the lateral meniscus level (tLCL) in ALL-intact and ALL-deficient knees.

Results

ALL was present in 27/34 (79%) of the knees. We found complete ALL in 13 cadavers bilaterally. ALL was absent bilaterally in three cadavers; it was present on one side and absent contralaterally in one cadaver. In ALL-intact knees, the average tLCL was 2.05 mm, whereas, in ALL-deficient knees, it was 2.57 mm. This difference in tLCL was statistically significant.

Conclusions

Our study adds new data to the recent voluminous research on ALL. We have examined the correlation between the thickness of ALL and LCL and documented alterations in the thickness of LCL in ALL-intact knees. These findings would help in designing reconstructive procedures for the combined ACL injury with ALRI.

## Introduction

Knowledge of the anatomy and biomechanics of the knee joint is crucial for understanding the various kinds of functional instabilities seen following a ligament injury. The most commonly injured and hence the most widely studied ligament of the knee joint is the anterior cruciate ligament (ACL) [1]. However, up to 25% of patients have varying grades of residual anterolateral rotational instabilities (ALRI) in the long term following surgical reconstruction of ACL [2]. Avulsion of anterolateral structures during the initial instability episode may produce a Segond fracture, which is considered pathognomonic for ACL tears, although this lesion may not always be present [3]. This indicates the stabilizing role of other structures in the knee joint.

The prevalence of concomitant lesions along with ACL injuries have been reported to be around 58%. These concomitant lesions lead to poorer functional outcomes and early-onset osteoarthritis compared to isolated ACL injuries [4]. The pivot shift phenomenon, which is used to assess ALRI, correlates with functional outcomes [2,3,5]. Residual pivot shift following ACL reconstruction has been thought to be due to concomitant anterolateral complex (ALC) and ACL injury in which only the ACL injury is addressed [5].

Lateral extra-articular reinforcement procedures have been used since the 1970s as a possible solution for persistent ALRI after primary ACL reconstruction [3,6]. There has been a renewed worldwide interest in defining the anterolateral corner of the knee following the publication of the precise anatomic details of the anterolateral ligament (ALL) [7-9]. Cadaveric as well as clinical studies have reported on the benefits of supplementing ALL reconstruction with ACL reconstruction in cases with concomitant injuries [10-12]. However, there are studies that claim that ALL reconstruction may be detrimental to knee function and produce early joint degeneration and lateral extra-articular reinforcement may be the preferred reconstruction option [13,14].

We undertook a cadaveric investigation with the objective of studying the anterolateral corner of 34 knees to define ALL and correlate the thickness of the lateral collateral ligament (tLCL) with that of ALL.

## Materials and methods

Study design: cross-sectional study

We studied 34 knees from 17 embalmed cadavers. All bodies had been donated for education and research to the Department of Anatomy, and this study was approved by the Institutional Human Ethics Committee. All the knees were free of gross deformities. These limbs had previously been dissected by undergraduate students in the Department of Anatomy, and in no case did the dissections affect the anterolateral structures of the knee. The cadaveric specimens were from eight females and nine males, with an average age of 82.8 years (range: 70-91 years); an equal number of knees from both sides were included.

Dissection of the anterolateral aspect of the knee joint was performed as per the description by Claes et al. [15]. To study ALL, the overlying superficial structure, i.e., the iliotibial band (ITB), was excised. Femoral and tibial attachment sites of ALL and its course were studied in detail with respect to adjoining soft tissue structures and bony landmarks. Standard digital calipers (capacity: 150 mm, accuracy: 0.01 mm) were employed. With the knee in internal rotation and 90-degree flexion, the following measurements were made: the length of ALL, the thickness of ALL (tALL), at the level of lateral inferior genicular artery (LIGA), and the thickness of LCL at the meniscal level (tLCL). After fully studying the anatomy of ALL, the ligament was excised and processed for histological examination.

Statistical analysis

We used Epi Info 7 software for statistical analysis. Frequency and percentage were used for summarizing categorical variables, whereas numerical variables were summarized using mean and standard deviation. We analyzed the difference in the thickness of LCL in ALL-intact and ALL-deficient knees. For this purpose, we used the t-test. A p-value of <0.05 was considered statistically significant.

## Results

We found ALL to be present in 27/34 (79%) of the knees. We found complete ALL in 13 cadavers bilaterally. ALL was absent bilaterally in three cadavers; it was present on one side and absent contralaterally in one cadaver. ALL was absent in three female cadavers bilaterally (six knees). The quantitative parameters of ALL are shown in Table 1.

**Table 1:** The quantitative parameters of ALL and LCL M: male; F: female; L: left; R: right; Y: yes; N: no; ALL: anterolateral ligament; LIGA: lateral inferior genicular artery; LCL: lateral collateral ligament

Cadaver number	Sex	Side	Presence of ALL	Thickness LIGA level, mm	LCL thickness, mm
1	M	L	Y	1.19	1.5
		R	Y	1.24	1.57
2	F	L	Y	1.06	1.84
		R	Y	0.77	1.16
3	F	L	Y	0.48	1.83
		R	Y	0.48	2.26
4	M	L	Y	1.15	1.4
		R	Y	0.5	1.73
5	M	L	Y	0.78	2.69
		R	Y	0.89	2.97
6	M	L	Y	0.93	1.93
		R	Y	1.07	2.36
7	M	L	Y	1.17	1.6
		R	Y	1.66	2.18
8	F	L	N		2.71
		R	N		2.4
9	F	L	N		2.64
		R	N		2.4
10	M	L	N		2.74
		R	Y	1.45	1.89
11	F	L	N		2.54
		R	N		2.57
12	F	L	Y	1.17	1.77
		R	Y	1.12	1.87
13	M	L	Y	1.25	2.2
		R	Y	1.28	2.45
14	M	L	Y	1.37	2.27
		R	Y	1.39	2.57
15	F	L	Y	1.15	2.47
		R	Y	1.14	2.71
16	F	L	Y	1.31	2.07
		R	Y	0.95	2.03
17	M	L	Y	1.02	2.15
		R	Y	1.24	1.95

Table 2 shows the correlation between the tLCL in ALL-intact and ALL-deficient knees. In ALL-intact knees, the average tLCL was 2.05 mm, whereas, in ALL-deficient knees, it was 2.57 mm. This difference was statistically significant (p-value: 0.0000001).

**Table 2:** Comparison between the thickness of LCL in ALL-deficient and ALL-intact knees ALL: anterolateral ligament; LCL: lateral collateral ligament

ALL-deficient knee (n=7)				ALL-intact knee (n=27)				P-value (by t-test)
Mean	Minimum	Maximum	Standard Deviation	Mean	Minimum	Maximum	Standard Deviation	0.0000001
2.57 mm	2.4 mm	2.74 mm	0.09	2.05 mm	1.16 mm	2.97 mm	0.85	

## Discussion

**Figure 1:**
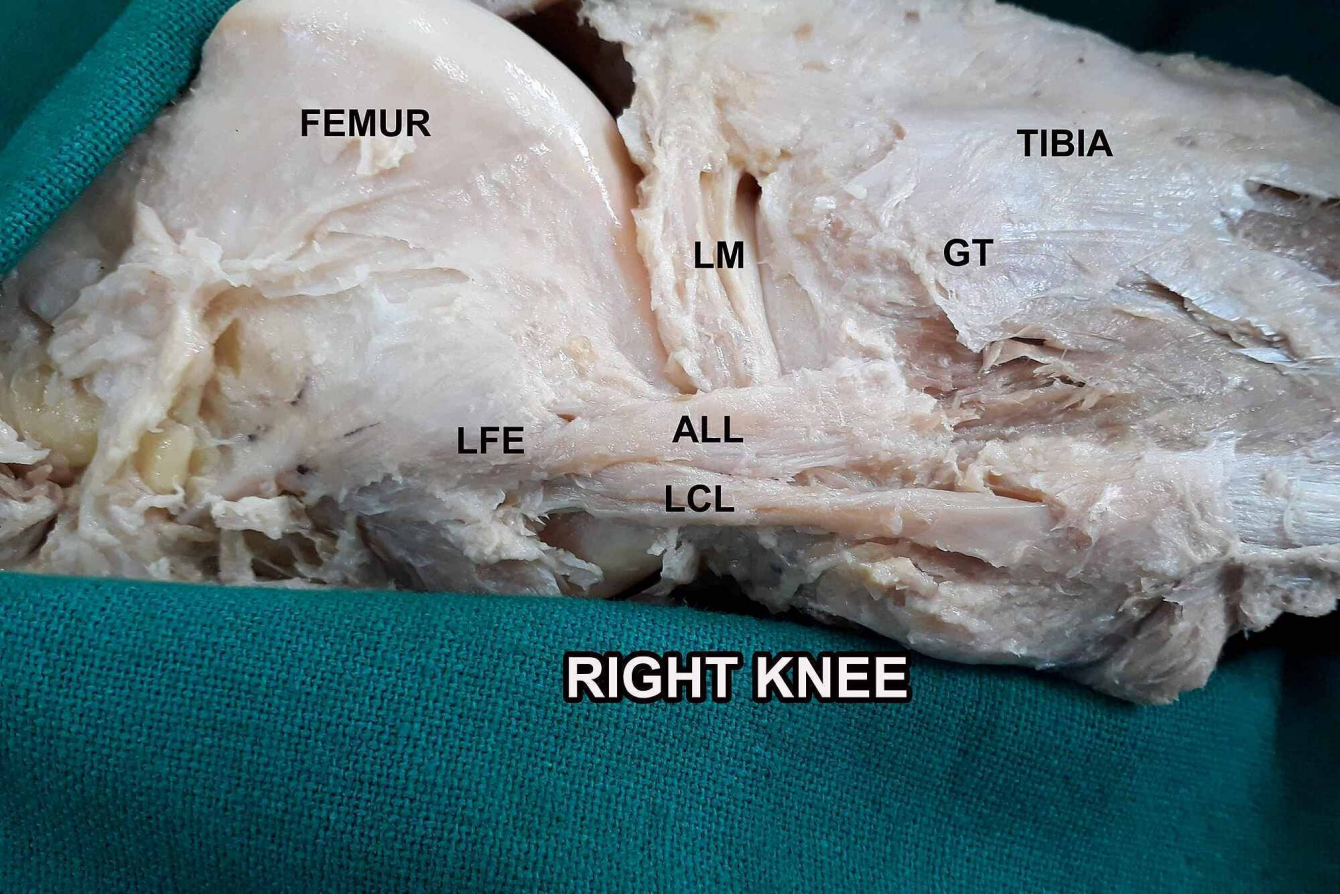
Anterior origin of ALL with respect to LCL (right knee) ALL: anterolateral ligament; LCL: lateral collateral ligament; GT: Gerdy's tubercle; LFE: lateral femoral epicondyle; LM: lateral meniscus

**Figure 2:**
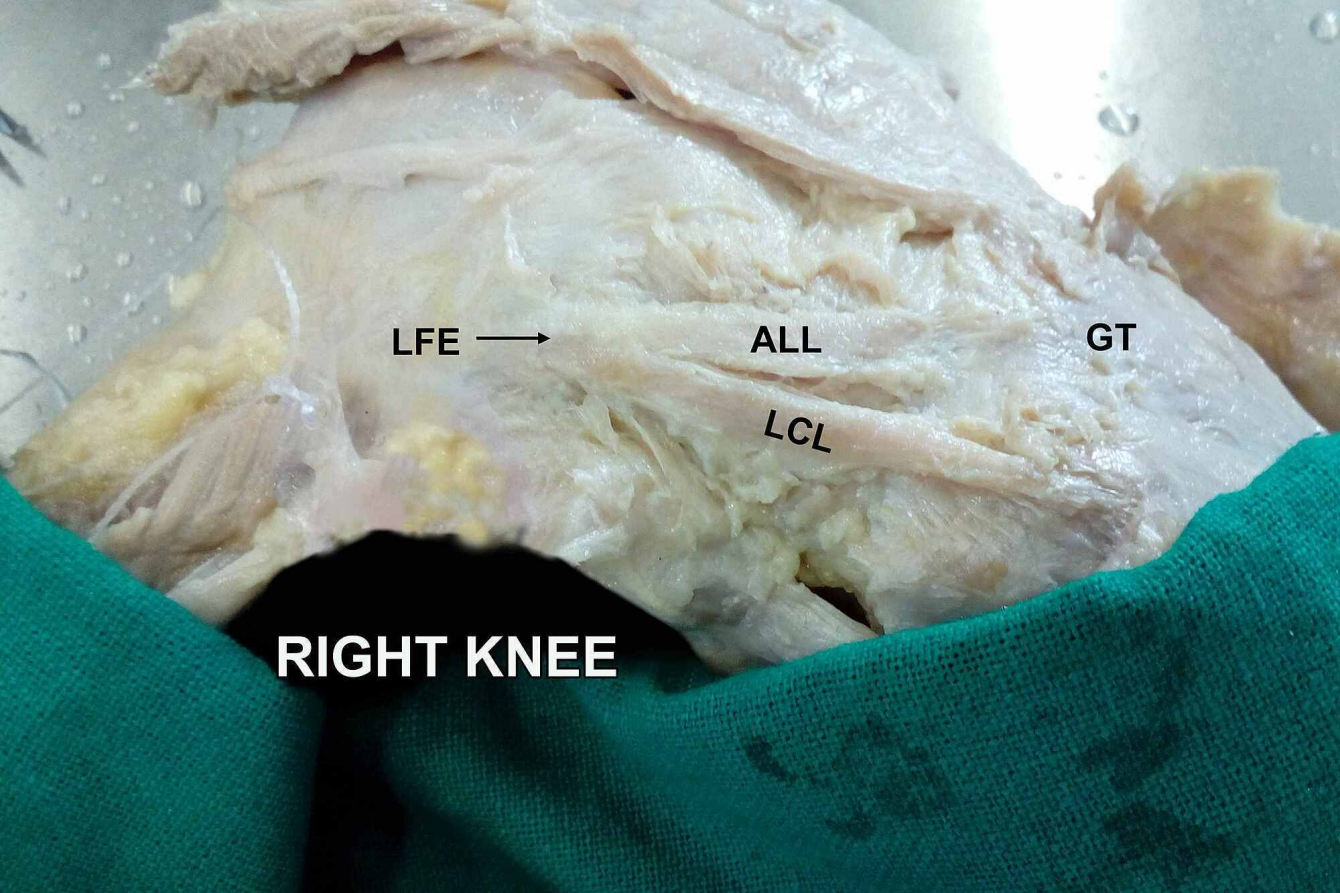
Common origin of ALL with LCL (right knee) ALL: anterolateral ligament; LCL: lateral collateral ligament; GT: Gerdy's tubercle; LFE: lateral femoral epicondyle

**Figure 3:**
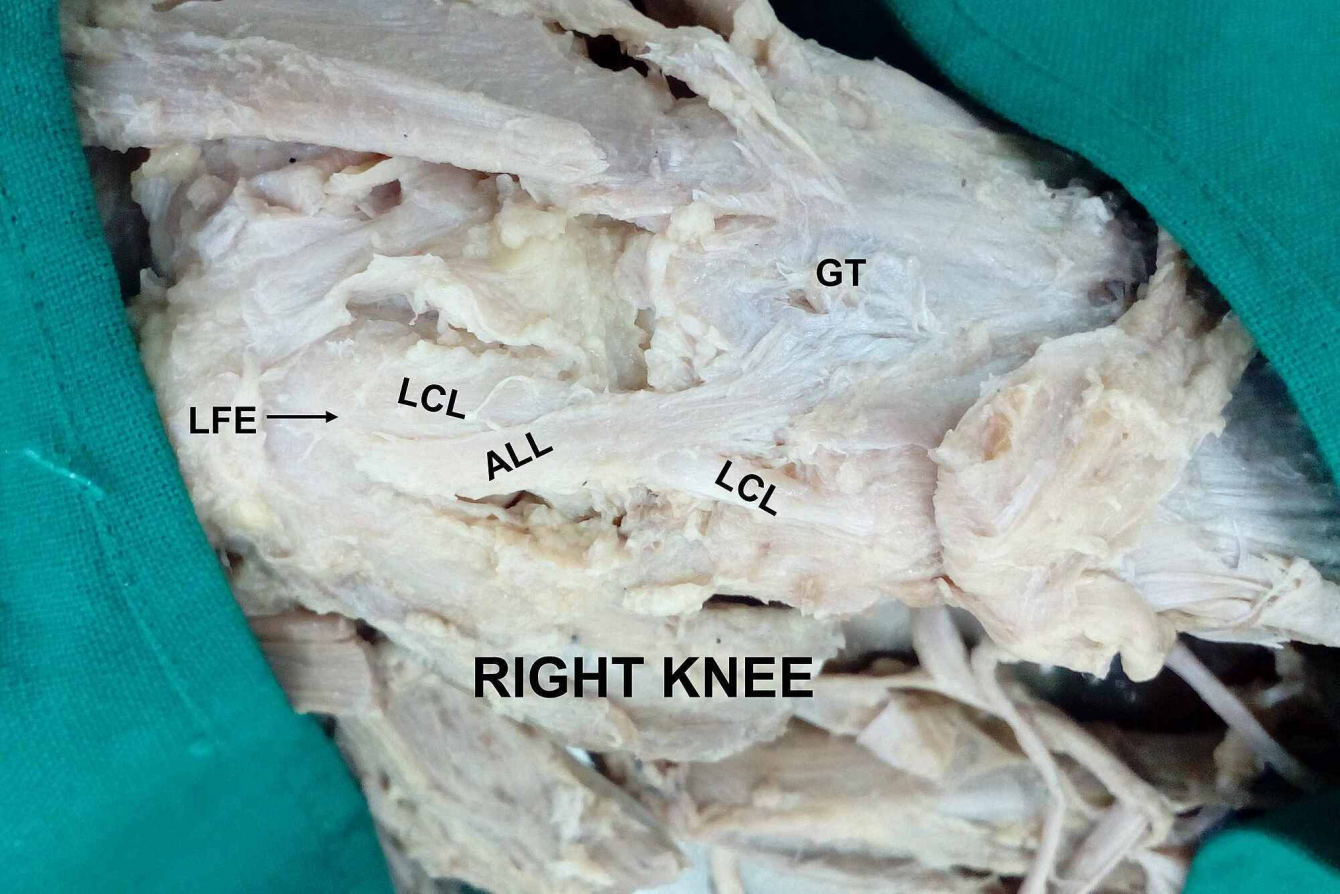
Posterior origin of ALL with respect to LCL (right knee) ALL: anterolateral ligament; LCL: lateral collateral ligament; GT: Gerdy's tubercle; LFE: lateral femoral epicondyle

Various cadaveric studies have demonstrated the presence of ALL in 50-100% of specimens [7-9,16-19]. In our study, ALL was present in 79% of the specimens (Figure 1, Figure 2, Figure 3). Taneja et al. in an imaging-based study identified the presence of the ALL in 51% of the knees; completely visible in 11% and partially visible in 40% of cases [16]. They could identify the tibial insertion in all cases. Kızılgöz et al. could recognize the entire ALL (femoral, meniscal, and tibial portions) in 82% of MR images [20]. These differences in identifying ALL in cadavers and imaging-based studies could be attributed to the differences in technique.

Our measurements are consistent with most cadaveric studies. The mean length of ALL in our study was 35.79 mm, which was in the range described by other authors [3,6,15].

Caterine et al. described the anatomical variations regarding the origin (lateral femoral epicondyle anterior-distal or proximal-distal to the LCL) and insertion (broad attachment approximately halfway between the midpoint of Gerdy’s tubercle and the LCL insertion onto the fibular head) of ALL [21]. Kosy et al. described the origin as antero-distal and postero-proximal in three and six cadaveric specimens respectively while in one its origin was the same as LCL [18]. In our dissections, 12 knees had the origin of ALL anterior to LCL (Figure 1), eight knees had a common origin of ALL and LCL (Figure 2), while two knees had the origin of ALL posterior to LCL (Figure 3).

A lot of recent work has been done in defining ALL and its role in knee stability; however, despite the close relation of ALL origin to that of LCL, LCL and ALL seem not to have been studied together. The ALL Expert Group consensus paper published in 2017 and the International ALC Consensus Group paper published in 2018 attempted to put into perspective several issues pertaining to ALL and ALC [22,23]. However, these papers were silent on the LCL of the knee in relation to ALL. We found relatively thin LCL in ALL-intact knees. The cadaveric study in fetuses by Toro-Ibarguen et al. identified ALL in 100% of specimens [19]. Based on our dissections, the cases of absent ALL, either in cadaveric or imaging studies, could represent a variation in the normal anatomy of the anterolateral corner of the knee. This is corroborated by our findings of a thicker LCL in ALL-deficient knees (Table 2). Therefore, we propose that ALL is just a normal variation of LCL.

Zens et al. studied the mechanical tensile properties of ALL and suggested that the entire ALC is responsible for the rotational stability of the knee rather than ALL alone [24]. According to them, ITB and anterolateral capsule have a more significant contribution compared to ALL in providing rotational stability. ITB is the primary restraint to tibial internal rotation above 30° knee flexion, and this increases with increasing knee flexion [25]. While ALL has a role in restraining tibial internal rotation, biomechanical data suggests that an ITB tenodesis based on Gerdy’s tubercle is more effective [26]. Based on our findings, we concur with Amis that in cases of ACL injury with ALRI, it may be more appropriate to view the entire ALC as the structure to be reconstructed along with ACL rather than ALL alone [25]. In fact, Kernkamp et al. and Inderhaug et al. have suggested that in these cases, compared to lateral extra-articular reinforcement procedures, anatomic ALL reconstruction might be biomechanically inappropriate and unsafe in restoring knee kinematics though there are no clinical studies to support it as yet [27,28].

Limitations

Biomechanical characterization of LCL in preventing ALRI in ALL-deficient knees would be of immense help in understanding kinematics in these knees. Connective tissue properties are altered by the fixation process in embalmed specimens, affecting the strength of fasciae and thereby precluding biomechanical characterization. However, there is no evidence that it alters the visible relationships.

## Conclusions

Our study adds new data to the recent voluminous research on ALL. We have examined the correlation between the thickness of ALL and LCL and documented alterations in the thickness of LCL in ALL-intact knees. These findings would help in designing reconstructive procedures for the combined ACL injury with ALRI.
